# Direct pesticide exposure of insects in nature conservation areas in Germany

**DOI:** 10.1038/s41598-021-03366-w

**Published:** 2021-12-16

**Authors:** Carsten A. Brühl, Nikita Bakanov, Sebastian Köthe, Lisa Eichler, Martin Sorg, Thomas Hörren, Roland Mühlethaler, Gotthard Meinel, Gerlind U. C. Lehmann

**Affiliations:** 1 Institute for Environmental Sciences Landau, University Koblenz Landau, Fortstraße 7, 76829 Landau, Germany; 2 Nature and Biodiversity Conservation Union (NABU), Charitéstraße 3, 10117 Berlin, Germany; 3 Leibniz Institute of Ecological Urban and Regional Development (IOER), Weberplatz 1, 01217 Dresden, Germany; 4 Entomological Society Krefeld (EVK), Marktstraße 159, 47798 Krefeld, Germany

**Keywords:** Biodiversity, Environmental impact, Environmental chemistry, Environmental monitoring, Entomology

## Abstract

In Germany, the decline of insect biomass was observed in nature conservation areas in agricultural landscapes. One of the main causal factors discussed is the use of synthetic pesticides in conventional agriculture. In a Germany-wide field study, we collected flying insects using Malaise traps in nature conservation areas adjacent to agricultural land. We used a multi-component chemical trace element analysis to detect 92 common agricultural pesticides in ethanol from insect traps sampled in May and August 2020. In total, residues of 47 current use pesticides were detected, and insect samples were on average contaminated with 16.7 pesticides. Residues of the herbicides metolachlor-S, prosulfocarb and terbuthylazine, and the fungicides azoxystrobin and fluopyram were recorded at all sites. The neonicotinoid thiacloprid was detected in 16 of 21 nature conservation areas, most likely due to final use before an EU-wide ban. A change in residue mixture composition was noticeable due to higher herbicide use in spring and increasing fungicide applications in summer. The number of substances of recorded residues is related to the proportion of agricultural production area in a radius of 2000 m. Therefore, a drastic pesticide reduction in large buffers around nature conservation areas is necessary to avoid contamination of their insect fauna.

## Introduction

After biomass reductions of almost 80% within 27 years were documented for Germany^1^, the decline of insects has received increased attention from researchers^2–5^ and the media^6,7^ in recent years. As a result, evaluations of the status of insect populations around the world were reviewed (^8^, and related publications in the same issue). These scientific and public discussions on insect decline were followed by political measures such as the “Action Programme for Insect Protection” of the Federal Government of Germany^9^ and changes in nature protection laws.

Insects play a crucial role within almost all trophic levels in terrestrial food webs. As primary consumers, herbivorous insects feed on plants and are then consumed by predators like other insects, spiders, amphibians, reptiles, birds and mammals as for example bats and shrews. The benefits of insect-mediated ecosystem functions and services such as nutrient cycling, soil formation, decomposition, water purification, biological pest control, pollination and food web interactions, which are all also critical to human health, were recently highlighted^10,11^. The decline of insects has become especially obvious in agricultural landscapes^1^ where the parallel decline of farmland birds has been recorded in Europe since the 1980s, and especially the reduction of insects as food for juvenile birds has been discussed over the past decades^12,13^.

Insecticides that are used to control “pest” insect species affect equally other non-target insects, many of them beneficial to the crop, not only in the treated agricultural fields but also in neighbouring habitats. The exposure and direct effects of neonicotinoid insecticides on pollinating insects such as honey bees has received special attention^14^. Wildflower pollen in field margins showed similar residue concentrations as treated oilseed rape pollen in the field^15^ and neonicotinoid concentrations were in the range of causing acute mortality in some insect species^16^. Additionally, herbicides are used to reduce *“weeds”* in fields that are food plants for insects^17^, therefore indirectly affecting higher trophic levels^18^. The role of herbicides in the decrease of insect food for grey partridge chicks was demonstrated in a field experiment almost 40 years ago^19^. A pan-European study on the biodiversity of plants, insects and birds in wheat fields identified pesticide applications as the main explanatory variable for reduced species numbers^20^. Especially for pollinators, the constant presence of mixtures of pesticides in the landscape has been suggested as a factor for their decline^16,21^. However, data on pesticide residues in the agricultural landscape remain scarce compared to the pesticide water monitoring that has been in place on country-wide scales for decades as a requirement of the EU Water Framework Directive^22^. The first data on pesticide residues measured in soils from agricultural fields have recently emerged^23–25^, and a landscape-scale assessment for exposure has been conducted for neonicotinoids^26^. Residues in pollen, a food source for honey bees *(Apis mellifera)* and other pollinating insects, have been monitored for years^27,28^, revealing bee exposure to multiple pesticides and seasonal changes of pesticide residue mixtures^29^. Pesticide residues on insects themselves have thus far been measured only for single substances in the context of bird and mammal risk assessment procedures in regulatory context [e.g.^30–32^]. In this study, we measured pesticide residues on insects in nature conservation areas embedded in agricultural landscapes, which allow a realistic assessment of pesticide exposure of flying insects for the first time.

The project DINA (Diversity of Insects in Nature protected Areas) investigates insect communities in 21 nature conservation areas belonging to the Natura2000 network in agricultural landscapes across Germany. Insects were collected in Malaise traps using ethanol as conservation fluid (for project details see^33^, SOM Table A1 and Fig. A1). The minimal distance to the cropping area bordering the nature conservation area was 25 m (see method section for details). Pesticide exposure of insects was directly measured by analysing pesticide residues present in the ethanol of the Malaise trap collecting bottles. This is a new methodological approach to provide qualitative data on pesticide contamination of insects that are alive.

The objectives of this study were to (1) evaluate exposure of the collected insects to residues of common current-use pesticides (CUPs), (2) to examine if there are seasonal differences in the pesticide residue mixtures and (3) to investigate the influence of conservation area size and proportion of surrounding agricultural area on insect pesticide exposure.

## Results and discussion

### Pesticide residues

Insects were collected in Malaise traps during two-week intervals, where pesticide residues from insect bodies were dissolved in the ethanol that was used to preserve the collected samples. Additionally, particles of plants, pollen, nectar or honeydew adhering to the insect bodies can be carriers of chemical pollution. Detected pesticide residues can therefore come from the insects and potentially attached particles. Under natural conditions of sunlight and warm temperatures, chemical stability of pesticide residues in the ethanol solution may have been affected by hydrolysis, for example, which could have caused the degradation of residues during the two-week collection intervals. Only flying insects that are alive can get into the Malaise traps, and therefore pesticide residues in the collected samples are assumed to represent sublethal levels to all trapped species. Additionally, insect collection was performed over an entire season and did not consider explicit spraying events. Therefore, the sampling we performed did not necessarily record maximum exposure levels that could represent lethal levels for individual species and substances. Hence, the quantification of pesticide amounts cannot be used for risk calculations. Instead we evaluate the presence of residues of CUPs on insects. Since detection is possible at low concentrations (see SOM Table A2) we obtained information on trace concentrations of the pesticide residues that insects were exposed to. It is safe to assume that the pesticide loads of insects were especially high following spraying events, and for individuals that were affected and consequently unable to fly. These insects were then not sampled in the Malaise traps.

Of the 92 target common CUPs, 47 were detected in the insect samples from 21 nature conservation areas from two sampling dates in May and August 2020: 13 herbicides, 28 fungicides and 6 insecticides. Additionally, metabolites of fipronil, an insecticide registered for biocidal use in the EU, were recorded at three locations. At the 21 sites, insects in the conservation areas were exposed to 16.7 pesticides on average, ranging from 7 to 27 substances. More fungicides than herbicides were recorded and, on average, insects were exposed to less than two insecticides (Table 1). This may in part reflect the application in arable crops where more fungicides than herbicides are applied and insecticides are used less frequently. On the other hand, as insecticides affect insects directly due to their high acute toxicity, exposure to insecticides results in mortality or sublethal effects that impair mobility, leading to an underestimation of insecticide residues in our samples.

**Table 1:** Number of CUP residues detected at 21 nature conservation areas across Germany and the resulting minimal, maximal and mean number of pesticide substances.

Abbreviation study site	Full name of study site	Herbicide residues	Fungicide residues	Insecticide residues	Sum pesticide residues
01_LUE	Lütjenholmer Heidedünen	5	3	1	9
02_RIE	Riedensee	10	11	1	22
03_KOO	Insel Koos	8	9	2	19
04_GEE	Geesower Hügel	10	9	2	21
05_MAL	Oderhänge Mallnow	5	7	2	14
06_WIS	Wisseler Dünen	6	13	1	20
07_BIS	Bislicher Insel	7	11	2	20
08_GIP	Gipskarstlandschaft Hainholz	5	6	1	12
09_POR	Porphyrlandschaft bei Gimritz	9	10	2	21
10_ZIE	Ziegenbuschhänge Niederau	8	17	2	27
11_WIP	Wipperdurchbruch	6	7	2	15
12_BOT	Bottendorfer Hügel	7	10	4	21
13_SBG	Schwellenburg	8	10	3	21
14_HOF	Hofberg	5	3	1	9
15_KOP	Koppelstein	4	3	0	7
16_DOE	Rheinhänge Dörscheider Heide	6	12	2	20
17_BRA	Brauselay	3	15	2	20
18_MIT	Mittelberg	5	5	1	11
19_IPF	Ipf	6	6	2	14
20_KUE	Kürnberg	6	9	2	17
21_MUE	Mühlhauser Halde	6	4	0	10
Minimum		3	3	0	7
Maximum		10	17	4	27
Mean		6.4	8.6	1.7	16.7

For study site locations and descriptions, (see (33) and SOM).

Insects at all 21 sites were exposed to residues of the herbicides metolachlor-S, prosulfocarb and terbuthylazine, and the fungicides azoxystrobin and fluopyram (Table 2). The presence of the six frequently detected herbicides can be explained by the high volume sold in 2019 (see SOM Table A3). They are among the 25 highest-ranking pesticides in terms of selling volume in Germany^34^. The same is true for the fungicide azoxystrobin. All other seven regularly detected fungicides were sold at lower volumes and their presence in the insect samples could be related to the high persistence of these fungicides, with soil half-lives reaching 500 d (bixafen), 484 d (boscalid) and 309 d (fluopyram). Only kresoxim-methyl, present in 10 sites, is not highly persistent in soil but has an affinity for the waxy plant cuticle, where it binds and accumulates^35,36^.

**Table 2:** CUP residues frequently recorded at the 21 sites. Only substances that were recorded in ≥ 10 sites are listed.

Herbicide	Presence	Fungicide	Presence	Insecticide		Presence
Metolachlor-S	21	Azoxystrobin	21	Thiacloprid	16	
Prosulfocarb	21	Fluopyram	21			
Terbuthylazine	21	Pyraclostrobin	17			
Dimethenamid	17	Bixafen	15			
Flufenacet	14	Boscalid	14			
Diflufenican	13	Fluazinam	14			
		Dimoxystrobin	13			
		Kresoxim-methyl	10			

The neonicotinoid insecticide thiacloprid was recorded on insects in 16 of the 21 nature conservation areas. Thiacloprid was banned in the EU for use in field applications from August 2020 onwards, however, the end of use (grace period) was set to 3rd February, 2021^37^. The high incidence of thiacloprid in our samples at many sites across Germany may therefore also reflect the last opportunity for farmers to use their remaining stocks. A ban could thus result in a greater impact to the ecosystem if parallel applications take place on a large scale. Hence, for potent pesticides which are banned from the market, it seems advisable to stop granting grace periods and instead destroy remaining stock rather than dispersing them into the environment despite knowledge of their high environmental risks.

On average, in spring (May) residues of 9.6 and in summer (August) 9.3 CUPs were recorded in individual ethanol samples of the three trapping locations in the conservation areas. The minimum number of pesticide residues of 3 (May, site Mülhauser Halde) and 2 (August, Mittelberg) and the maximum of 16 (May, Bottendorfer Hügel) and 18 (August, Wisseler Dünen) were all from samples closer to the centre of the nature protection area, furthest away from adjacent agricultural fields.

### Seasonality of CUP exposure

**Figure 1:**
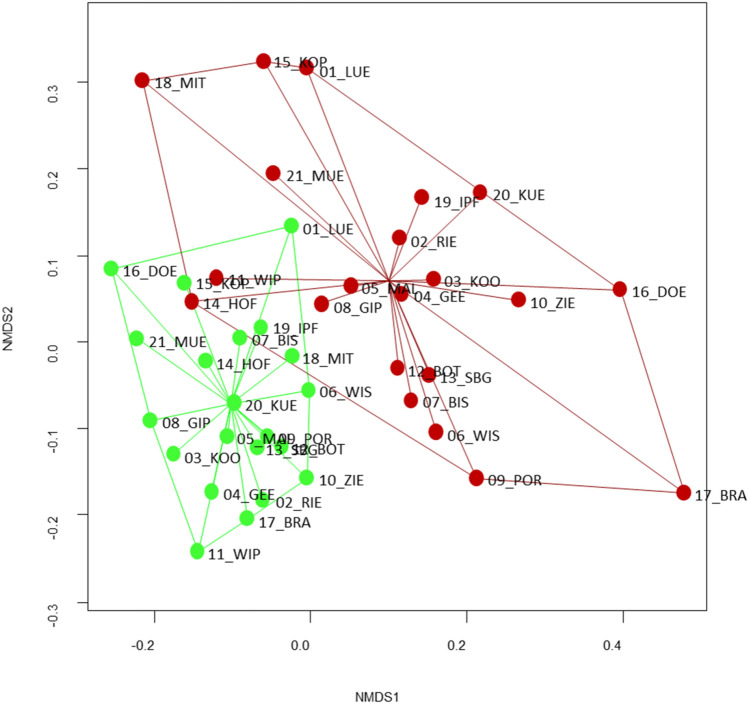
CUP mixtures in May (green) and August (red) analysed with NMDS. The position of each location was determined by the composition of pesticide residues found in the ethanol samples. The closer data points are located in the ordination space, the more similar are their composition of pesticides. For abbreviations see Table 1.

The total number of CUP residues recorded on insects was similar for the two sampling intervals with 32 substances in May and 35 in August. However, a higher number of herbicide residues was recorded in May (13) compared to August (9), whereas for fungicides the reverse was the case [August (23), May (14)]. The number of detected insecticide residues was similar, with three and five substances recorded in May and August, respectively. This resulted in a different set of pesticide residue mixtures, driven by seasonality (Fig. 1). Mixtures in May, dominated by herbicides, were more similar to each other than the August mixtures, which contained more fungicides. The extreme positions of the NMDS analysis in August with Brauselay and Mittelberg are driven by the number of fungicide residues recorded. Brauselay is the only site where vineyards bordered the study area. Wine growing in Germany requires frequent fungicide applications.

On the substance level, residues of the herbicides prosulfocarb, metolachlor-S, dimethenamid-P were recorded in more than half of the sites at both sampling intervals, whereas terbuthylazine was frequently present in May but not in August, and flufenacet was detected more frequently later in the year. Fungicide residues of fluopyram, azoxystrobin and boscalid were common in both sampling intervals, but pyraclostrobin, bixafen and dimoxystrobin were characteristic for May samples and fluazinam and kresoxim-methyl for the August samples (SOM Table A4). Although more residues of fungicides were recorded in August, this did not result in an increase in the number of fungicides that are found at many sites. Thirteen out of the 23 fungicides that were recorded in August were detected comparatively sporadic in samples from one to three sites. For insecticides, only thiacloprid was frequently noted, and the remaining substances acetamiprid, dimethoate, tebufenozide, and indoxacarb were found in May, whereas chlorantraniliprole and indoxacarb were recorded in August. The observed patterns reflect the agricultural practice of using herbicides in spring and early summer to establish crops such as cereals, oilseed rape and maize, and fungicides later in the year to control fungal diseases that increase with warmer temperatures.

In addition to pesticide applications, seasonality has a direct effect on insect communities that change in composition from spring to autumn^38–40^. Because of shifts in insect community composition and pesticide application schemes, the mixture of pesticide residues present in insect samples changes throughout the year. Thus, it is likely that a finer time resolution than the selected two sampling intervals could reveal additional pesticide residues for the exposure of insects in conservation areas in the agricultural landscape.

### Influence of surrounding agricultural production area

**Figure 2:**
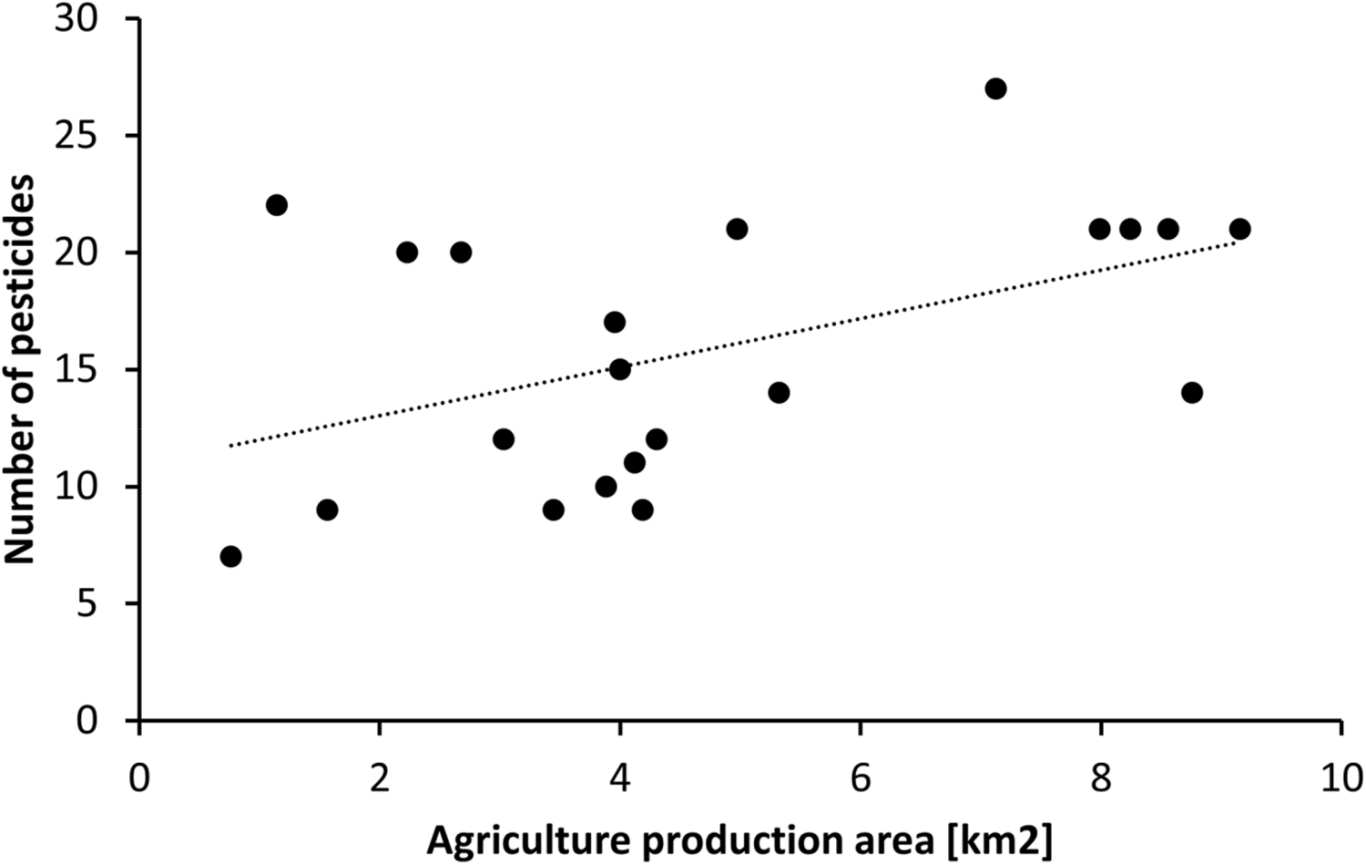
The number of CUP residues per site detected in insect/ethanol samples increased with the area of agriculture in a radius of 2000 m around the trapping positions (Pearson correlation coefficient = 0.48, *p* = 0.029).

Our data demonstrate that insects collected with Malaise traps in the nature conservation areas are exposed to pesticides applied in the surrounding agricultural landscape, where various crops are grown and are treated with a variety of pesticides. As the flight range of aerial insects fluctuates from less than one hundred meters to kilometres (for examples from the literature see SOM Table A5), it is not only the neighbouring arable field that may act as a source of contamination. A correlation analysis of the area of arable fields in the surrounding landscape (buffered from 500 to 3500 m) and number of pesticide residues recorded in the insect-trapping ethanol revealed a best fit for a radius of 2000 m around the center of the trapping positions in the conservation area (Fig. 2, all 21 sites, Pearson correlation coefficient = 0.48, *p* = 0.029). The site Brauselay differed from all nature conservation areas as vineyards were bordering the nature conservation area. Wine growing is a permanent crop characterised by high fungicide use on a comparable small area. When removing Brauselay from the analysis significance increased further (Pearson correlation coefficient = 0.60, *p* = 0.005; for further details, see SOM Fig. A2 and Table A6). Hence, pesticide residues on insects collected in the nature conservation areas are not only a result of applications on crops in the direct vicinity, but also from pesticide use in a larger area within the agricultural landscape around the conservation areas.

Based on the correlation between pesticides and surrounding arable land, a generalized linear mixed model (GLMM) was applied to model the number of detected pesticide residues as a function of landscape factors (amount of agricultural production area, amount of nature conservation area and amount of FFH area in a 2000 m radius) and biomass of insects collected by the Malaise traps, with the study sites included as random effects (Table 3). Neither the area of the nature conservation area nor the FFH area nor biomass of collected insects was related to the number of pesticides recorded in ethanol samples. Only the agricultural production area in a 2000 m vicinity had a significant (*p* < 0.022) impact on the number of pesticide residues found in the ethanol samples. A higher proportion of arable land was related to a higher number of CUP residues measured on insects in the nature conservation area. The significance of this relationship increased further (*p* = 0.008) when excluding the wine growing site Brauselay (SOM Table A7. Analysing the two sampling intervals separately showed a highly significant relationship of detected numbers of pesticides and agricultural production area in May (*p* < 0.001) but not in August (SOM Table A8).

**Table 3:** Results of GLMM.

	Estimate	Std. Error	z value	Pr( >|z|)
Formula: pesticides ~ agricultural production area + nature conservation area + FFH area + biomass + (1|area)				
(Intercept)	2.246	0.311	7.218	** < 0.001**
Agricultural production area	0.066	0.029	2.298	**0.022**
Nature conservation area (km^2^ )	0.012	0.099	0.119	0.906
FFH area (km^2^ )	0.014	0.083	0.165	0.869
Biomass	< 0.001	< 0.001	0.982	0.326

The number of detected CUPs was analysed in regard to amount of agricultural production area (arable crops, wine and fruit growing), nature conservation area and FFH area within a 2000 m radius and the biomass of insects collected by the Malaise traps (fixed effects), while the study sites (area) function as random effects. Significant values in bold.

Insecticides are causing direct, lethal effects on insect populations, or reducing their foraging activity or reproduction. Herbicides can also have direct effects on insects, causing mortality, but generally affect insects indirectly by reducing wild plant or “weed” cover in agricultural fields, and as a result, diminish food availability for insects^17^). Fungicides not only affect fungal diseases but can also be directly toxic to insects or other invertebrates^41^). An example is the fungicide fluopyram, which was detected in all nature conservation areas of our study. Fluopyram is also marketed and used as a nematicide in many crops^42^ and consequently also has the potential to affect invertebrates directly. The detected mixtures of multiple pesticides, indirect effects and effects at field relevant application rates are not considered in the environmental risk assessment carried out for pesticide regulation in Europe, and the risk of pesticides for biodiversity is, therefore, underestimated^43^.

## Conclusion

Insects collected in nature conservation areas showed to have been exposed to a variety of pesticide residues and mixtures that changed throughout the year. These insects must have been exposed to pesticides in the surrounding agricultural landscape, where conventional farming using synthetic pesticides predominates.

Currently legislation was implemented in Germany that bans the use of herbicides and bee toxic insecticides in nature conservation areas, although applications are still allowed in larger, surrounding FFH areas. However, to protect insects in nature conservation areas, it is not only mandatory to stop pesticide use there, but it is necessary to also reduce synthetic pesticide applications especially in the vicinity, preferably to form a protective buffer zone. Our analysis of differing landscape surroundings from 500 to 3500 m, representing potential flight ranges of insects, revealed that the number of pesticides detected is related to the agricultural production area within a radius of around 2000 m. Protective buffers to reduce pesticide contamination of insects should therefore be established in the range of multiple 100 m and not few 10 m. Yet, local structural features, agricultural practice in the surrounding as well as composition of insect communities present need to be taken into account in the planning. A 2000 m buffer around all nature conservation areas would affect around 30% of Germany's total arable land. Although this is a large proportion it is within the area of transformation suggested in the EU Green Deal, that aims for 25% of agricultural land under organic farming by 2030^44^. We propose that future transformation in land management could be specifically targeted around nature conservation areas to form the required buffer zones of organic agriculture where no synthetic pesticides are applied. In Germany, bans for synthetic pesticide use could be extended in first steps to the larger FFH areas. Buffer zones are an integral part of the concept of sustainable use of pesticides in the European Union in relation to the contamination of surface water bodies, but not for terrestrial nature conservation areas. However, it is recognised that “pesticides can be particularly dangerous in very sensitive areas, such as Natura2000 sites”^45^. The legislation further reminds that “When pesticides are used, appropriate risk management measures should be established and low-risk pesticides as well as biological control measures should be considered in the first place.” Landscape planning concerns habitats and their characteristic species designated as a “Special Area of Conservation” within the European Union Natura2000 network of protected areas (for our study see SOM Table A1). Until now, nature conservation planning has unfortunately largely excluded pesticides from risk assessment and implementation. In a first step we especially need data transparency about pesticide applications inside and next to nature conservation areas for a meaningful planning.

The presented approach of residue analysis of ethanol samples from insects collected with Malaise traps provided reliable results documenting a realistic exposure of insects towards pesticide mixtures. Any ethanol as a by-product from insect sampling can be used for analysis and no costly extraction steps are needed. Maximum residue loads on insects are not measured since collecting is not conducted parallel to spraying events and only sublethal levels are present on insects collected in traps. However, the obtained qualitative information of low residue concentrations provides a better estimate of the realistic exposure of insects with CUP mixtures in agricultural as well as other landscapes. This information can be used to include multiple pesticides in environmental risk assessment approaches. Additionally, residue analysis of ethanol from insect sampling has the potential to be implemented in monitoring schemes for biodiversity which are currently set up by authorities. With an increase in locations and sampling time we could be able to get a more detailed view of realistic CUP exposure of insects at a landscape scale, which is also necessary to monitor the effectiveness of future transformations in agricultural landscapes, aiming for reductions of synthetic pesticides and increases of organic farming.

We showed that insect populations in conservation areas are exposed to residue mixtures of multiple CUPs. So far, no data are available which evaluate the effects of chronic exposure of terrestrial insect populations or communities to realistic mixtures of multiple pesticides. The topic has received attention in aquatic ecotoxicology^46,47^ and studies with mixtures of realistic pesticide concentrations have shown lethal effects on amphibian tadpoles^48^ and invertebrates over multiple weeks^49^. These data of aquatic systems call for caution and it seems that toxicity data gained from short-term exposure towards single pesticides cannot be used to rule out the risk of a further reduction of insect population size in the long-run. A recent meta-analysis showed that assuming additive effects, the risk of pesticide exposure may underestimate the interaction on bee mortality, which is synergistic in most cases and standard risk assessment therefore fails to protect insects^50^. Hence, it is justified to apply the precautionary principle and call for a partial or total reduction of pesticide exposure of insects in nature conservation areas and their surrounding.

With the identified decline of insects in agricultural landscapes, it is vital to at least preserve threatened insect populations as parts of local biodiversity in the nature conservation areas situated in this landscape. Urgent action is required, as tipping points for already small populations of certain insect species may have been reached (e.g.^51^). Expanding protected areas or adding an effective buffer zone of untreated or reduced synthetic pesticide use in surrounding crops are potential options to diminish the risk of pesticide exposure and the resulting impacts on these insects and further biodiversity.

## Methods

### Ethics statement

Permission to collect insects in the 21 nature conservation areas was granted by the relevant authorities of the different federal states of Germany.

### Study sites

21 nature conservation areas (German: Naturschutzgebiete NSG) in the Natura2000 Network in Germany were selected to cover different habitat types, ranging from dry sites such as dunes, dry grasslands and heaths, meadows and shrub lands, to wet sites such as reedbeds, and to represent a wide geographical spread (SOM Table A1, Fig. A1^52^). All sites bordered agricultural fields. More information on study sites and background can be found in a description of the DINA project^33^. Transects consisting of five Malaise traps starting from arable fields with annual crops such as cereals, maize, sugar beet, and in one site (Brauselay) vineyards, were established into the conservation areas. The study sites varied in size from 0.12 km^2^ (Kürnberg) to 15.74 km^2^ (Insel Koos) with an average of 2.81 km^2^. The first trap was situated in an arable field next to the nature conservation area and the second was placed at the border of arable land and conservation area. For our analysis, we used the samples from three Malaise traps established inside the nature conservation areas at 25 m and 50 m distance to bordering crops, as well as an additional trap that was set closer to the core of the nature conservation area with a distance from the field ranging from 61 to 127 m.

### Insect collection and sample processing

Flying insects were collected using the identical, standardised sampling design of Malaise trapping used in the only German long-term study on insect communities evaluating biomass (^1,53^ SOM Fig. A4). Traps were always oriented with the opening towards the South and insects were sampled in light-protected collecting bottles filled with 96% ethanol, as the sampled insects were used for metabarcoding. Trapping started in mid-April and ended in October 2020. Ethanol of the fourteen-day sampling interval from mid to end of May (14/5–30/5/2020) and from the end of July to the beginning of August (24/7–7/8/2020) were used for pesticide analysis. Sampling was synchronized across Germany, varying maximally four days between the 21 sites. Insects were collected in ethanol and remained there under environmental conditions for approximately 14 days. The wet biomass of insects was measured at the Entomological Society Krefeld (EVK) using a standardized protocol (1). A 100 ml sample of the trapping ethanol was shipped to iES (institute for Environmental Sciences) Landau for pesticide analysis.

### Pesticide analysis

An aliquot of 50 ml of ethanol from each sample was completely evaporated under a gentle nitrogen stream. After evaporation, the sample was dissolved with 1 ml of methanol (LC–MS grade, > 99.9%, Honeywell, Seelze, Germany), vortexed (60 s), filtered (13 mm HPLC syringe filter, 0.2 µm, PTFE, hydrophobic) and directly injected in the high performance liquid chromatography-tandem mass spectrometry instrument (HPLC–MS/MS; LC: Agilent Technologies LC 1260 Infinity series, MS/MS: Agilent Technologies 6495C, Waldbronn, Germany) for analysis (details on analytical method SOM Table A9/1–3). We analysed for 92 common CUPs in Germany based on records of the Julius-Kühn-Institute published in the PAPA database^54^. Pesticides used in winter wheat, oilseed rape, maize, potato and wine in 2016 and 2017 were selected. Frequently-used herbicides and fungicides as well as all insecticides were included in the analysis. Additionally, newer insecticides such as chlorantraniliprole were included together with pesticides that were regularly detected in German small streams^55^.

Method quantitation limits (MQL) and method detection limits (MDL) of all targeted analytes (see SOM Table A2 for full list of analytes) ranged from 0.0004 to 0.186 µg/l and from 0.0002 to 0.0614 µg/l, respectively. Pesticides detected below the MDL were classified as non-detected, all others as detected. As the ethanol was not cooled in the field, and break down of pesticides is therefore likely, a quantification of pesticides is not informative. Consequently, pesticides are solely classified as present or absent in a sample. CUP residues of the three samples of a site are summed to indicate the exposure of insects at each nature conservation area. Maximum and minimum values of individual samples are also presented.

### Landscape analysis

The geodata set of the Federal Agency for Nature Conservation (BfN) on protected areas 2018 was used for the delineation of the 21 selected nature conservation areas and for the calculation of their areas^52^. The agricultural landscape around the study sites was evaluated using the Digital Land Cover Model Germany (LBM-DE) 2018 of the Federal Agency for Cartography and Geodesy (BKG) in a GIS approach^56^. The LBM-DE provides an area-wide description of the landscape by topographic objects for Germany. Land use and land cover are geometrically delineated. Arable land can be identified in the LBM-DE as a combination of the land use "arable land" (B211) and the land cover “arable land” (N211). In addition, in the LBM-DE, vineyard areas were derived from the land use "viticulture" (B221) and the land cover "vineyard" (N211), and orchard areas from the land use "fruit and soft fruit" (B222) and the land cover "orchards" (N211). Arable land, vineyards and fruit orchards were then summarised as agricultural production area, where pesticides are used to grow crops. To obtain information on the proportion of the agricultural production area within a radius of 500–3500 m, the transect consisting of traps 3 to 5 was buffered by 500 m, 1000 m, 1500 m, 2000 m, 2500 m, 3000 m, and 3500 m (SOM Fig. A4). Distances were estimated using daily flight ranges of insects collected with Malaise traps (see SOM Table A5). For the agricultural production area identified within these radii, the area and proportion of the total area of the radius was calculated.

### Statistical analysis

The statistical analysis was conducted using Rx64 4.0.1^57^. To test the data for normal distribution, the Shapiro–Wilk test was applied and correlation analysis according to Pearson was performed to analyse the relation between CUP residues and the amount of agricultural production area in the different radii.

The R package “lme4”^58^ was used to run a generalized linear mixed model (GLMM) to analyse the number of pesticides present at a location in relation to the size of the protected area and the amount of agricultural production area in its 2000 m vicinity (fixed effects) while the study sites were included as random effects.

Non-metric multidimensional scaling (NMDS) was applied to depict dissimilarities between the study sites and their respective pesticide composition, with special emphasis on differences between samples from May and August. The R packages “vegan”^59^ and “ggplot2”^60^ were applied for NMDS analysis.

## Supplementary Information

Supplementary Information.
