# Supplementary material for: Direct pesticide exposure of insects in nature conservation areas in Germany
Source: Sci Rep. 2021 Dec 16;11:24144. doi: 10.1038/s41598-021-03366-w (PMC8677746; doi:10.1038/s41598-021-03366-w)
Supplement: Supplementary file 1 — Supplementary Information. [file 41598_2021_3366_MOESM1_ESM.pdf]

Supplementary Information (SI)

Direct pesticide exposure of insects in nature conservation areas in Germany

Carsten A. Brühl[1*], Nikita Bakanov[1], Sebastian Köthe[2], Lisa Eichler[3], Martin Sorg[4], Thomas Hörren[4], Roland Mühlethaler[2], Gotthard Meinel[3], Gerlind U.C. Lehmann[2]

[1]Institute for Environmental Sciences Landau, University Koblenz Landau, Fortstraße 7,76829 Landau, Germany

[2]Nature and Biodiversity Conservation Union (NABU), Charitéstraße 3, 10117 Berlin, Germany

[3]Leibniz Institute of Ecological Urban and Regional Development (IOER), Weberplatz 1, 01217 Dresden, Germany

[4]Entomological Society Krefeld (EVK), Marktstraße 159, 47798 Krefeld, Germany

Tables

Table A1 Description of the 21 study sites.

Nr.	Location	State	FFH-ID *1	FFH Area [ha] *1	NSG-ID *2	NSG Area [ha] *2	Altitude [m a.s.l.]	X-Coord. [°] *3	Y-Coord. [°] *3	Protected habitat types (the transect is crossing) *4	Natura2000-code *4
1	**Lütjenholmer Heidedünen**	SH	1320-302	313	37570	18	5	90.624	546.991	Dry sand heaths with *Calluna* and *Genista*	2310
										Northern Atlantic wet heaths with *Erica tetralix*	4010
2	**Riedensee**	MV	1836-301	113	33182	120	2	116.835	541.527	Xeric sand calcareous grasslands	6120
3	**Insel Koos**	MV	1747-301	60406	33165	1574	2	134.019	541.615	Lowland hay meadows	6510
4	**Geesower Hügel**	BB	2752-301	82	30009	39	20	143.918	532.391	Xeric sand calcareous grasslands	6120
										Sub-pannonic steppic grasslands	6240
5	**Oderhänge Mallnow**	BB	3552-306	305	30118	305	15	144.675	524.740	Xeric sand calcareous grasslands	6120
										Sub-pannonic steppic grasslands	6240
6	**Wisseler Dünen**	NW	4203-301	71	35714	79	15	63.012	517.699	Inland dunes with open *Corynephorus* and *Agrostis* grasslands	2330
7	**Bislicher Insel**	NW	4305-301	1002	36965	1053	20	65.114	516.483	Rivers with muddy banks with Bidention-Vegetation	3270
										Hydrophilous tall herb fringe communities of plains	6430
8	**Gipskarstlandschaft Hainholz**	NI	4226-301	1327	33265	641	20	102.659	516.870	Lowland hay meadows	6510
9	**Porphyrlandschaft bei Gimritz**	ST	4437-302	819	38460	288	100	118.478	515.668	Sub-pannonic steppic grasslands	6240
										Siliceous rock with pioneer vegetation	8230
10	**Ziegenbuschhänge bei Oberau**	SN	4847-301	112	37981	20	155	135.577	511.947	Lowland hay meadows	6510
11	**Wipperdurchbruch**	TH	4631-302	6869	38140	672	285	110.497	513.190	Semi-natural (semi-)dry grasslands and scrubland facies on calcareous substrates (Festuco-Brometalia)	6210

Table A1 (cont.) Description of the 21 study sites.

Nr.	Location	State	FFH-ID *1	FFH Area [ha] *1	NSG-ID *2	NSG Area [ha] *2	Altitude [m a.s.l.]	X-Coord. [°] *3	Y-Coord. [°] *3	Protected habitat types (the transect is crossing) *4	Natura2000-code *4
12	**Bottendorfer Hügel**	TH	4634-303	133	38143	134	195	114.070	513.143	Calaminarian grasslands of the *Violetalia calaminariae*	6130
										Sub-pannonic steppic grasslands	6240
13	**Schwellenburg**	TH	4931-301	89	38111	23	210	109.567	510.330	Rupicolous calcareous or basiphilic grassland of the *Alysso-Sedion albi*	6110
										Sub-pannonic steppic grasslands	6240
14	**Hofberg**	TH	5327-305	260	38319	43	430	102.285	506.975	Rupicolous calcareous or basiphilic grassland of the *Alysso-Sedion albi*	6110
										Semi-natural dry grasslands and scrubland facies on calcareous substrates (Festuco-Brometalia)	6210
15	**Koppelstein - Helmestal**	RP	5711-301	4551	37188	87	160	76.321	502.905	Semi-natural (semi-)dry grasslands and scrubland facies on calcareous substrates (Festuco-Brometalia)	6210
										Lowland hay meadows	6510
16	**Rheinhänge Dorscheider Heide**	RP	5711-301	4551	37186	626	220	77.488	501.158	European dry heaths	4030
										Siliceous rock with pioneer vegetation of the Sedo-Scleranthion	8230
17	**Brauselay**	RP	5809-301	16273	37136	14	240	71.848	501.437	Subcontinental peri-pannonic scrub	40A0
										Stable xerothermophilous formations with *Buxus sempervirens* on rock slopes (Berberidion)	5110
										Silicate rocks with pioneer grassland	8230
										Tilio-Acerion forests of slopes, screes and ravines	9180

Table A1 (cont.) Description of the 21 study sites.

Nr.	Location	State	FFH-ID *1	FFH Area [ha] *1	NSG-ID *2	NSG Area [ha] *2	Altitude [m a.s.l.]	X-Coord. [°] *3	Y-Coord. [°] *3	Protected habitat types (the transect is crossing) *4	Natura2000-code *4
**18**	**Mittelberg**	BW	7319-341	853	30714	45	460	88.853	487.456	Semi-natural (semi-)dry grasslands and scrubland facies on calcareous substrates (Festuco-Brometalia)	6210
**19**	**Ipf**	BW	7327-341	3363	30585	60	650	103.585	488.713	*Juniperus communis* formations on calcareous grasslands	5130
										Semi-natural dry grasslands and scrubland facies on calcareous substrates (Festuco-Brometalia)	6210
**20**	**Kürnberg**	BW	7427-341	990	30689	12	510	102.485	486.440	*Juniperus communis* formations on calcareous grasslands	5130
										Semi-natural (semi-)dry grasslands and scrubland facies on calcareous substrates (Festuco-Brometalia)	6210
**21**	**Mühlhauser Halde**	BW	7916-311	3678	31149	52	720	85.864	480.557	*Juniperus communis* formations on calcareous grasslands	5130
										Semi-natural (semi-)dry grasslands and scrubland facies on calcareous substrates (Festuco-Brometalia)	6210

*1 https://www.bfn.de/themen/natura-2000/natura-2000-gebiete/steckbriefe.html#c33722

*2 https://geodienste.bfn.de/schutzgebiete?lang=de&layers=NSG

*3 Reference system DHDN, Gauß-Krüger Zone 3.

*4 EC European Commission (2013): EUR 28 Interpretation Manual of European Union habitats. https://ec.europa.eu/environment/nature/legislation/habitatsdirective/docs/Int_Manual_EU28.pdf

Table A2 Method limits of quantitation (MQL) and method limits of detection (MDL) for all 92 analytes.

Substance	CAS	MQL (µg/l)	MDL (µg/l)
Herbicides			
2,4-D	94-75-7	0.0798	0.0264
Aminopyralid	150114-71-9	0.0058	0.0018
Bentazone	25057-89-0	0.0028	0.001
Bromoxynil	1689-84-5	0.0042	0.0014
Carfentrazone-ethyl	128621-72-7	0.1194	0.0394
Chloridazon	1698-60-8	0.0012	0.0004
Chlortoluron	15545-48-9	0.0006	0.0002
Clomazone	81777-89-1	0.0018	0.0006
Diflufenican	83164-33-4	0.0006	0.0002
Dimethenamid-P	163515-14-8	0.0006	0.0002
Ethofumesate	26225-79-6	0.0242	0.008
Flazasulfuron	104040-78-0	0.005	0.0016
Florasulam	145701-23-1	0.046	0.0152
Flufenacet	142459-58-3	0.0032	0.001
Fluroxypyr	69377-81-7	0.043	0.0142
Flurtamone	96525-23-4	0.0006	0.0002
Foramsulfuron	173159-57-4	0.0068	0.0022
Isoproturon	34123-59-6	0.0008	0.0002
Lenacil	2164-08-1	0.03	0.01
MCPA	94-74-6	0.0796	0.0262
Metamitron	41394-05-2	0.006	0.002
Metazachlor	67129-08-2	0.0032	0.001
Metobromuron	3060-89-7	0.003	0.001
Metolachlor-S	87392-12-9	0.0014	0.0004
Metsulfuron-methyl	317815-83-1	0.0154	0.0052
Napropamide	15299-99-7	0.0004	0.0002
Pendimethalin	40487-42-1	0.0058	0.002
Picloram	1918-02-1	0.1044	0.0344
Propaquizafop	111479-05-1	0.0008	0.0002
Propyzamide	23950-58-5	0.0046	0.0016
Prosulfocarb	52888-80-9	0.001	0.0004
Quinmerac	90717-03-6	0.0006	0.0002
Quizalofop-P	94051-08-8	0.1862	0.0614
Terbuthylazine	5915-41-3	0.0064	0.0022
Tribenuron-methyl	101200-48-0	0.0124	0.004
Tritosulfuron	142469-14-5	0.096	0.0316

Table A2. (cont.) Method limits of quantitation (MQL) and method limits of detection (MDL) for all 92 analytes.

Substance	CAS	MQL (µg/l)	MDL (µg/l)
Fungicides			
Azoxystrobin	131860-33-8	0.0018	0.0006
Benalaxyl	71626-11-4	0.001	0.0004
Bixafen	581809-46-3	0.0008	0.0002
Boscalid	188425-85-6	0.0052	0.0016
Cyazofamid	120116-88-3	0.0542	0.0178
Cyflufenamid	180409-60-3	0.0298	0.0098
Cymoxanil	57966-95-7	0.008	0.0026
Cyprodinil	121552-61-2	0.0092	0.003
Difenconazole	119446-68-3	0.0042	0.0014
Dimethomorph	110488-70-5	0.0044	0.0014
Dimoxystrobin	149961-52-4	0.0006	0.0002
Epoxiconazole	135319-73-2	0.0032	0.001
Fenoxycarb	79127-80-3	0.0024	0.0008
Fenpropimorph	67564-91-4	0.0062	0.002
Fluazinam	79622-59-6	0.0048	0.0016
Fludioxonil	131341-86-1	0.0224	0.0074
Fluopicolid	239110-15-7	0.0034	0.0012
Fluopyram	658066-35-4	0.0016	0.0006
Iprovalicarb	140923-17-7	0.0016	0.0006
Kresoxim methyl	143390-89-0	0.0058	0.002
Mandipropamid	374726-62-2	0.007	0.0022
Metalaxyl-M	70630-17-0	0.0016	0.0006
Metconazole	125116-23-6	0.0008	0.0002
Metrafenone	220899-03-6	0.0014	0.0004
Myclobutanil	88671-89-0	0.0018	0.0006
Paclobutrazol	76738-62-0	0.0032	0.001
Penconazole	66246-88-6	0.0024	0.0008
Pencycuron	66063-05-6	0.0008	0.0002
Picoxystrobin	117428-22-5	0.0008	0.0002
Prochloraz	67747-09-5	0.006	0.002
Propamocarb	24579-73-5	0.001	0.0004
Proquinazid	189278-12-4	0.0014	0.0004
Prothioconazole	178928-70-6	0.1118	0.0368
Pyraclostrobin	175013-18-0	0.001	0.0004
Pyrimethanil	53112-28-0	0.0034	0.0012
Spiroxamine	118134-30-8	0.0024	0.0008
Tebuconazole	107534-96-3	0.0132	0.0044
Trifloxystrobin	141517-21-7	0.0018	0.0006

Table A2. (cont.) Method limits of quantitation (MQL) and method limits of detection (MDL) for all 92 analytes.

Substance	CAS	MQL (µg/l)	MDL (µg/l)
Insecticides, Acaricides			
Acetamiprid	135410-20-7	0.001	0.0004
Avermectin B1a	65195-55-3	0.13	0.0428
Chlorantraniliprole	500008-45-7	0.0014	0.0004
Clothianidin	210880-92-5	0.007	0.0024
Dimethoate	60-51-5	0.0006	0.0002
Etofenprox	80844-07-1	0.001	0.0004
Fenpyroximate	111812-58-9	0.001	0.0004
Flonicamid	158062-67-0	0.0066	0.0022
Hexythiazox	78587-05-0	0.0016	0.0006
Imidacloprid	138261-41-3	0.0056	0.0018
Indoxacarb	173584-44-6	0.0034	0.0012
Methiocarb	2032-65-7	0.0024	0.0008
Pirimicarb	23103-98-2	0.0014	0.0004
Pymetrozine	123312-89-0	0.0012	0.0004
Spinosad A	131929-90-7	0.001	0.0004
Spinosad D	131929-63-0	0.0074	0.0024
Tebufenozide	112410-23-8	0.0018	0.0006
Thiacloprid	111988-49-9	0.0004	0.0002
Thiamethoxam	153719-23-4	0.0066	0.0022
Biocides (metabolites)			
Fipronil desulfinyl	205650-65-3	0.0034	0.0012
Fipronil	120068-37-3	0.0128	0.0042
Fipronil sulfone	120068-36-2	0.0044	0.0014

Table A3. Soil half-lives and volume sold for use in 2019 for most commonly detected CUPs (H = Herbicide, F = Fungicide, I = Insecticide). Use volume for 2019 was extracted from BVL 2021, rank was calculated without carbon dioxide. T a.i. = metric tons of active ingredient.

		**presence**	**soil half life**			**persistence**	**use 2019 (Germany)**		
**group**	**substance**	**in sites** (of 21)	**DT50 typical**	**DT50 lab**	**DT50 field**	**interpretation**	**in t a.i**	**rank**	**remarks**
H	Metolachlor-S	21	90	15	21	moderate	573.6	14	
H	Prosulfocarb	21	11.9	11.9	9.8	no	966.5	7	
H	Terbuthylazine	21	72	72	21.8	moderate	972.9	6	
H	Dimethenamid-P	17	11	12.1	15.8	no	594.0	13	
H	Flufenacet	14	19.7	19.7	39	no	650.4	11	
H	Diflufenican	13	94.5	94.5	64.6	moderate	303.7	21	
F	Azoxystrobin	21	78	84.5	180.7	persistent	330.7	19	
F	Fluopyram	21	309	309	118.8	persistent	82,8	65	
F	Pyraclostrobin	17	41.9	41.9	33.3	moderate	74,5	67	
F	Bixafen	15	500	500	254	very persistent	84.4	62	
F	Boscalid)	14	484.4	484.4	254	very persistent	103.6	48	
F	Fluazinam	14	124	124	25.9	persistent	102.3	50	
F	Dimoxystrobin	13	210	210	22.9	persistent	15.4	123	
F	Kresoxim-Methyl	10	16	0.87	1	no	12.2	133	mixture product
I	Thiacloprid	16	0.88	0.88	8.1	no	93.8	56	final year for use

H = herbicide, F = fungicide, I = insecticide

Table A4. Most common pesticide substances recorded at the 21 sites. Only substances that were recorded in ≥ 10 sites are shown.

May

Herbicide	presence	Fungicide	presence	Insecticide	presence
Prosulfocarb	21	Azoxystrobin	21	Thiacloprid	14
Terbuthylazine	21	Fluopyram	21		
Metolachlor-S	17	Pyraclostrobin	17		
Dimethenamid-P	11	Bixafen	15		
Diflufenican	10	Dimoxystrobin	13		
		Boscalid	12		

August

Herbicide	presence	Fungicide	presence	Insecticide	presence
Prosulfocarb	20	Fluopyram	21	Thiacloprid	11
Metolachlor-S	16	Azoxystrobin	17		
Dimethenamid-P	13	Fluazinam	14		
Flufenacet	12	Boscalid	12		
		Kresoxim-Methyl	10		

Table A5 Examples of daily flight activity ranges of different insect groups. Potentially insects of these species and / or groups are collected with Malaise traps.

Species	Insect group	Daily flight range	Source
Lasioglossum. calceatum	Solitary bee	>1000 m	Beil *et al.* 2008
Hylaeus punctulatissimus	Solitary bee	1100 m (Maximum)	Zurbuchen *et al.* 2010
Chelostoma rapunculi	Solitary bee	1275 m (Maximum)	Zurbuchen *et al.* 2010
Hoplitis adunca	Solitary bee	1400 m (Maximum)	Zurbuchen *et al.* 2010
Aglais urticae	Butterfly	150-1210 m	Cant *et al.* 2005
Hesperia comma	Butterfly	50-1070 m	Hill *et al.* 1996
Musca domestica	Fly	Up to 7000 m	Nazni *et al.* 2005

Sources:

Beil, M., Horn, H. & Schwabe, A. Analysis of pollen loads in a wild bee community (Hymenoptera: Apidae) – a method for elucidating habitat use and foraging distances. *Apidologie* **39** 456–467 (2008).

Cant, E. T., Smith, A. D., Reynolds, D. R., & Osborne, J. L. Tracking butterfly flight paths across the landscape with harmonic radar. *Proc. Roy. Soc. B: Biol. Sci.* **272** 785-790 (2005).

Hill, J. K., Thomas, C. D., & Lewis, O. T. Effects of habitat patch size and isolation on dispersal by Hesperia comma butterflies: implications for metapopulation structure. *J. Anim. Ecol.* **65** 725-735 (1996).

Nazni, W. A., *et al.* Determination of the flight range and dispersal of the house fly, *Musca domestica* (L.) using mark release recapture technique. *Trop. Biomed.* **22** 53-61 (2005).

Zurbuchen, A., Cheesman, S., Klaiber, J., Müller, A., Hein, S., & Dorn, S. Long foraging distances impose high costs on offspring production in solitary bees. *J. Anim. Ecol.* **79** 674-681 (2010).

Table A6. Results of Pearson's correlation analysis for comparisons between the number of found pesticide residues and the amount of agricultural production area (arable crops, wine and fruit growing, in km^2) in the respective radii (500-3500 m, see also Figure A3). Significant values in bold. A. All sites. B. The study site Brauselay was excluded from the analysis. Brauselay is the only site where vineyards are bordering the nature conservation area and not arable crops. Wine growing in Germany is using multiple fungicide application per season and differs from arable crops.

A: All 21 sites.

Radius	**t**	**df**	**p**	**cor**
500 m	1.015	19	0.323	0.227
1000 m	1.336	19	0.197	0.293
1500 m	1.394	19	0.179	0.305
2000 m	2.362	19	**0.029**	0.476
2500 m	1.269	19	0.220	0.279
3000 m	1.159	19	0.261	0.257
3500 m	1.919	19	0.070	0.403

B: *20 sites – Wine growing site Brauselay excluded.*

**Radius**	**t**	**df**	**p**	**cor**
500 m	1.291	18	0.213	0.291
1000 m	1.719	18	0.103	0.376
1500 m	1.883	18	0.076	0.406
2000 m	3.211	18	**0.005**	0.604
2500 m	1.766	18	0.094	0.384
3000 m	1.583	18	0.131	0.350
3500 m	2.499	18	**0.022**	0.508

Table A7 Results of GLMM when excluding the wine growing site Brauselay. The number of detected CUPs was analysed in regard to amount of agricultural production area and the size of nature conservation area and FFH area in a radius of 2000 m of the transect center as well as biomass of insects collected by the malaise traps (fixed effects), while the study sites (area) function as random effects. Significant values in bold.

Formula:

pesticides ~ agricultural production area + nature_protected_area + FFH_area + biomass + (1 | area)

**Combined data**	**Estimate**	**Std. Error**	**z value**	**Pr(>|z|)**
(Intercept)	2.199	0.311	7.079	**<0.001**
Agricultural production area	0.079	0.030	2.674	**0.008**
Nature conservation area (km^2)	0.025	0.099	0.257	0.797
FFH area	0.018	0.082	0.216	0.829
Biomass	0.001	0.001	0.560	0.576
**May**	**Estimate**	**Std. Error**	**z value**	**Pr(>|z|)**
(Intercept)	1.611	0.308	5.224	**< 0.001**
Agricultural production area	0.111	0.030	3.664	**< 0.001**
Nature conservation area (km^2)	-0.042	0.105	-0.400	0.689
FFH area	0.052	0.088	0.588	0.557
Biomass	0.003	0.002	1.417	0.156
**August**	**Estimate**	**Std. Error**	**z value**	**Pr(>|z|)**
(Intercept)	1.916	0.390	4.906	**< 0.001**
Arable land	0.063	0.041	1.547	0.122
Nature conservation area (km^2)	0.083	0.135	0.612	0.541
FFH area	-0.007	0.111	-0.061	0.951
Biomass	<-0.001	0.002	-0.058	0.954

Table A8 Results of GLMM including all sites with separate analysis of May and August samples. The number of detected CUPs was analysed in regard to amount of agricultural production area and the size of nature conservation area and FFH area in a radius of 2000 m of the transect center as well as biomass of insects collected by the malaise traps (fixed effects), while the study sites (area) function as random effects. Significant values in bold.

Formula:

pesticides ~ agricultural production area + nature conservation area + FFH area + biomass + (1 | area)

**May**	**Estimate**	**Std. Error**	**z value**	**Pr(>|z|)**
(Intercept)	1.624	0.036	5.309	**< 0.001**
Agricultural production area	0.106	0.029	3.607	**< 0.001**
Nature conservation area (km^2)	-0.046	0.105	-0.438	0.661
FFH area	0.048	0.087	0.552	0.581
Biomass	0.004	0.029	1.747	0.081
**August**	**Estimate**	**Std. Error**	**z value**	**Pr(>|z|)**
(Intercept)	2.054	0.377	5.455	**< 0.001**
Agricultural production area	0.041	0.038	1.095	0.274
Nature conservation area (km^2)	0.067	0.135	0.499	0.618
FFH area	-0.015	0.112	-0.135	0.892
Biomass	<0.001	0.002	0.141	0.888

Pesticide analysis.

LC-MS/MS analysis.

Table A8-1 and A8-2 show details on analytical method conditions. For a selective detection of the selected substances, the mass spectrometer was set to multiple reaction monitoring (MRM). For quantitation and confirmation of the selected substances, at least two MRM transitions, the retention time (RT) (cf. Table A8-3), and the signal-to-noise ratio of >10 were used, except for proquinazid with one MRM transition only. The LC-MS/MS data was processed using Agilent MassHunter Workstation (Quantitative analysis for QQQ version 10, Agilent Technologies, Inc., Santa Clara CA, USA).

Calibration standards.

The substances in ethanol samples were quantified using matrix-matched external calibration (calibration standards, n=7). Matrix-matched standards were prepared using blank ethanol, which was processed in the same manner as the ethanol field samples. Briefly, an aliquot of 50 ml of ethanol was completely evaporated under a gentle nitrogen stream. After evaporation, the sample was dissolved with 1 ml of methanol (LC-MS grade, >99.9%, Honeywell, Seelze, Germany), vortexed (60 s), filtered (13 mm HPLC syringe filter, 0.2 µm, PTFE, hydrophobic). Several tens of millilitres of the blank ethanol solution were prepared to be used for preparation of matrix-matched calibration standards. The majority of substances for calibration standards were mixtures of neat analytical-quality standards, which were purchased from Restek, USA. The substances avermectin B1a, Dimethoate, fenpyroximate, hexythiazox, napropamid, proquinazid, and tebufenozide as individual standards were from Sigma-Aldrich, Seelze, Germany and LGC, Standards, UK.

QA/QC.

To compensate for response drift bracketing calibrations were used, i.e. one set of calibration standards run before and one after a batch of samples (n=20). Samples were evaluated on average of both calibrations after both calibrations were measured. The quality control program consisted of solvent blanks, method (ethanol) blanks and quality control samples (fortified blank ethanol) at three concentration levels (representing the lower, middle and upper levels of the calibration range) and were measured with each batch of samples (n=20). Additionally, calibration standards were checked for linearity and the retention time of the substances for deviation after each batch of samples (n=20).

Method detection limits (MDLs) and Method quantitation limits (MQLs)

For determination of MDLs and MQLs a spiking level 2 times the estimated MDL of each of the selected substances was selected. Thereafter, blank ethanol sample (50 ml; n=7 each substance) was spiked with the selected level, evaporated to dryness under a gentle nitrogen stream, dissolved in 1 ml of methanol, filtered and analysed. The results of the analysis were used for the calculation of the MDLs and MQLs.

The MQLs and MDLs were calculated using the linear regression parameters from the substance calibration curves with

$$MDL_S = 3.3 \times S / a \text{ and } MQL_S = 10 \times S / a$$

where S is the sample standard deviation of the replicate (n=7) spiked ethanol sample analyses and a is the slope of the regression of calibration curve. The values 3.3 and 10 represent the signal-to-noise ratio. The individual MQLs and MDLs are shown in Table A2, whereby the values represent substance concentrations in µg/l divided by 50 (concentration factor of the 50 ml of the evaporated ethanol).

Table A9/1. Procedures of analytical analysis: Agilent 1260 Infinity II UHPLC conditions.

Parameter	Value	
Column	Agilent ZORBAX Eclipse Plus C18, 3x150 mm, 2.7 µ	
Column temperature	45 °C	
Injection volume	1 µL	
Speed	Draw, 200 µl/min Eject: 400 µL/min	
Needle wash	12 seconds	
Mobile phase	A) 98 % Water with 4 mM ammonium formate + 0.1 % acetic acid + 2% methanol<br>B) 98 % methanol with 4 mM ammonium formate + 0.1 % acetic acid + 2% water	
Flow rate	0.45 mL/min	
Gradient programme	Time	B%
	0	2
	1.0	50
	4.0	65
	14.0	100
	20.0	100
	20.1	2
Stop time	20.1 minutes	
Post time	3 minutes	

Table A9/2. Procedures of analytical analysis: Agilent 6495C MS parameters for positive and negative ion method.

Parameter	Value
Mass Spectrometer	Agilent 6495C with electrospray ionization (ESI) operated in multiple reaction monitoring mode
Ionization mode	Positive / Negative
Gas temperature	250 °C; 11 L/min
Nebulizer	38 psi
Sheath gas	350 °C; 12 L/min
Capillary voltage	3,000 V / 3000 V
Delta EMV	200
High pressure iFunnel RF	150 V / 150 V
Low pressure iFunnel RF	60 V / 60 V
MS1 and MS2 resolution	Unit

Table A9/3. MRM transitions of analytes in positive and negative ion mode method.

Substance	Quantifier					Qualifiers			
	**Retention time (minutes)**	**Precursor ion**	**Product ion**	**Ion polarity**	**Collision energy (eV)**	**Precursor ion**	**Product ion**	**Ion polarity**	**Collision energy (eV)**
Herbicides									
2,4-D	9.83	221.0	161.0	Negative	12	219.0	161.0	Positive	12
Aminopyralid	9.84	207.0	134.0	Positive	32	207.0	107.0	Positive	52
Bentazone	8.42	239.0	132.0	Positive	29	239.0	197.0	Positive	21
						241.0	199.0	Positive	4
Bromoxynil	9.13	274.0	79.0	Negative	37	275.7	79.0	Negative	8
						275.7	81.0	Negative	8
Carfentrazone-ethyl	13.35	412.0	346.1	Positive	22	412.0	277.1	Positive	30
						412.0	366.1	Positive	15
Chloridazon	7.15	222.0	77.0	Positive	36	222.0	92.0	Positive	24
						222.0	65.1	Positive	44
Chlortoluron	9.02	213.1	72.0	Positive	8	213.1	46.1	Positive	8
Clomazone	10.61	240.1	125.0	Positive	20	240.1	89.1	Positive	56
Diflufenican	14.72	395.1	266.0	Positive	24	395.1	238.0	Positive	40
						395.1	246.0	Positive	36
Dimethenamid-P	11.34	276.1	244.0	Positive	13	276.1	168.1	Positive	25
Ethofumesate	10.96	304.1	121.1	Positive	30	287.0	121.0	Positive	10
						304.1	241.0	Positive	4
Flazasulfuron	10.18	408.1	182.1	Positive	28	408.1	139.1	Positive	40
						408.1	83.0	Positive	40
Florasulam	7.40	360.0	129.0	Positive	28	360.0	144.0	Positive	20
						360.0	192.0	Positive	12
Flufenacet	12.43	364.1	152.1	Positive	16	364.1	194.2	Positive	4
						364.1	124.1	Positive	36
Fluroxypyr	8.31	255.0	209.0	Positive	19	255.0	181.0	Positive	29
Flurtamone	10.77	334.1	227.0	Positive	44	334.1	275.2	Positive	20
						334.1	303.2	Positive	20
Foramsulfuron	8.97	453.0	181.9	Positive	46	453.0	255.1	Positive	21
Isoproturon	10.11	207.2	46.1	Positive	8	207.2	72.1	Positive	5
Lenacil	10.09	235.1	82.1	Positive	36	235.1	55.1	Positive	44
MCPA	9.88	201.0	141.0	Negative	12	201.0	143.0	Negative	12
Metamitron	6.78	203.1	77.0	Positive	32	203.1	175.1	Positive	12
						203.1	104.1	Positive	20
Metazachlor	10.14	278.1	134.2	Positive	15	278.1	105.1	Positive	44
						278.1	79.1	Positive	60
Metobromuron	9.81	259.0	170.0	Positive	20	259.0	148.0	Positive	15
Metolachlor-S	12.87	284.1	176.2	Positive	29	284.1	134.1	Positive	37
Metsulfuron-methyl	8.22	382.1	199.0	Positive	20	382.1	167.0	Positive	12
Napropamide	12.37	272.2	129.0	Positive	12	272.2	171.1	Positive	16
						272.2	58.1	Positive	28

Table A9/3. (cont.). MRM transitions of analytes in positive and negative ion mode method.

Substance	Quantifier					Qualifiers			
	Retention time (minutes)	Precursor ion	Product ion	Ion polarity	Collision energy (eV)	Precursor ion	Product ion	Ion polarity	Collision energy (eV)
Pendimethalin	16.16	282.1	212.1	Positive	4	282.1	194.1	Positive	16
						282.1	43.2	Positive	32
Picloram	5.70	240.9	194.8	Positive	25	240.9	167.9	Positive	37
						240.9	140.8	Positive	53
Propaquizafop	15.45	444.1	100.1	Positive	15	444.1	371.0	Positive	10
Propyzamide	11.84	256.0	190.0	Positive	10	256.0	173.0	Positive	20
						256.0	145.0	Positive	36
Prosulfocarb	15.10	252.1	91.2	Positive	20	252.1	128.2	Positive	8
						252.1	65.1	Positive	60
Quinmerac	7.19	222.0	204.0	Positive	18	222.0	141.0	Positive	38
						222.0	114.0	Positive	56
Quizalofop-P	12.80	345.1	162.9	Positive	40	345.1	91.1	Positive	28
						345.1	180.0	Positive	60
Terbuthylazine	11.28	230.1	174.1	Positive	15	230.1	96.1	Positive	24
						230.1	68.0	Positive	40
Tribenuron-methyl	9.20	396.1	181.0	Positive	20	396.1	180.9	Positive	16
						396.1	155.0	Positive	8
Tritosulfuron	9.98	446.0	195.0	Positive	23	446.0	145.0	Positive	54
Fungicides									
Azoxystrobin	10.18	404.1	372.1	Positive	8	404.1	344.1	Positive	24
						404.1	329.1	Positive	32
Benalaxyl	13.57	326.2	148.1	Positive	27	326.2	91.1	Positive	48
						326.2	121.1	Positive	32
Bixafen	12.96	414.0	394.0	Positive	20	414.0	266.0	Positive	20
Boscalid	11.19	343.0	307.1	Positive	16	343.0	271.2	Positive	32
						343.0	272.1	Positive	32
Cyazofamid	12.38	325.0	108.0	Positive	8	325.0	44.1	Positive	28
						325.0	261.0	Positive	4
Cyflufenamid	12.96	413.1	295.1	Positive	10	413.1	359.1	Positive	10
						413.1	223.0	Positive	20
Cymoxanil	6.93	199.1	128.0	Positive	4	199.1	110.9	Positive	12
						199.1	83.0	Positive	20
Cyprodinil	11.83	226.1	76.9	Positive	50	226.1	92.9	Positive	40
						226.1	65.1	Positive	56
Difenconazole	14.39	406.1	251.0	Positive	20	406.1	188.0	Positive	40
						406.1	337.0	Positive	10
Dimethomorph	11.17	388.1	301.1	Positive	20	388.1	139.0	Positive	36
						388.1	165.1	Positive	32
Dimoxystrobin	13.19	327.2	205.1	Positive	12	327.2	116.0	Positive	20
						327.2	58.0	Positive	40
Epoxiconazole	12.21	330.1	121.0	Positive	16	330.1	101.2	Positive	52
						330.1	95.0	Positive	60

Table A9/3. (cont.). MRM transitions of analytes in positive and negative ion mode method.

Substance	Quantifier					Qualifiers			
	Retention time (minutes)	Precursor ion	Product ion	Ion polarity	Collision energy (eV)	Precursor ion	Product ion	Ion polarity	Collision energy (eV)
Fenoxycarb	12.96	302.1	88.0	Positive	29	302.1	256.1	Positive	8
						302.1	116.0	Positive	17
Fenpropimorph	10.95	304.3	57.1	Positive	32	304.3	130.0	Positive	25
						304.3	98.1	Positive	32
Fluazinam	15.28	462.9	416.0	Negative	10	462.9	398.0	Negative	9
Fludioxonil	11.08	247.0	126.0	Negative	32	247.0	169.0	Negative	32
						247.0	152.0	Negative	32
Fluopicolid	11.44	382.9	172.9	Positive	20	382.9	144.9	Positive	56
						382.9	109.1	Positive	60
Fluopyram	12.18	397.0	145.0	Positive	61	397.0	172.9	Positive	41
						397.0	207.9	Positive	37
Iprovalicarb	12.06	321.2	119.0	Positive	16	321.2	116.1	Positive	16
						321.2	91.1	Positive	56
Kresoxim methyl	13.24	314.1	222.1	Positive	10	314.1	267.0	Positive	0
						314.1	206.0	Positive	0
Mandipropamid	10.99	412.1	328.1	Positive	8	412.1	356.1	Positive	4
						412.1	125.0	Positive	40
Metalaxyl-M	9.84	280.2	220.1	Positive	10	280.2	160.1	Positive	20
						280.2	45.1	Positive	36
Metconazole	13.85	320.1	70.1	Positive	24	320.1	125.0	Positive	48
						320.1	43.0	Positive	60
Metrafenone	13.96	409.1	209.1	Positive	8	409.1	166.1	Positive	40
						409.1	226.9	Positive	16
Myclobutanil	11.72	289.1	70.1	Positive	16	289.1	89.1	Positive	60
						289.1	125.1	Positive	32
Paclobutrazol	11.31	294.1	70.1	Positive	16	294.1	125.2	Positive	36
						294.1	57.2	Positive	20
Penconazole	13.18	284.1	70.1	Positive	15	284.1	123.1	Positive	56
						284.1	159.0	Positive	30
Pencycuron	14.17	329.1	125.1	Positive	24	329.1	217.9	Positive	12
						329.1	89.1	Positive	60
Picoxystrobin	12.79	368.1	205.2	Positive	4	368.1	145.0	Positive	20
						368.1	115.0	Positive	56
Prochloraz	13.74	376.0	308.0	Positive	4	376.0	70.1	Positive	24
						376.0	265.9	Positive	12
Propamocarb	5.64	189.2	102.0	Positive	12	189.2	144.0	Positive	8
						189.2	74.0	Positive	24
Proquinazid	17.10	372.9	289.0	Positive	20				
Prothioconazole	14.04	344.0	154.0	Positive	40	344.0	125.0	Positive	40
						344.0	102.0	Positive	40
Pyraclostrobin	13.47	388.1	193.8	Positive	8	388.1	164.1	Positive	12
						388.1	163.1	Positive	20

Table A9/3. (cont.). MRM transitions of analytes in positive and negative ion mode method.

Substance	Quantifier					Qualifiers			
	**Retention time (minutes)**	**Precursor ion**	**Product ion**	**Ion polarity**	**Collision energy (eV)**	**Precursor ion**	**Product ion**	**Ion polarity**	**Collision energy (eV)**
Pyrimethanil	10.73	200.1	82.0	Positive	5	200.1	106.9	Positive	20
						200.1	77.1	Positive	40
Spiroxamine	11.09	298.3	144.1	Positive	16	298.3	100.1	Positive	32
						298.3	41.1	Positive	60
Tebuconazole	13.19	308.1	70.0	Positive	40	308.1	124.9	Positive	47
						308.1	57.1	Positive	28
Trifloxystrobin	14.30	409.1	186.0	Positive	12	409.1	145.0	Positive	52
						409.1	116.0	Positive	24
Insecticides, Acaricides									
Acetamiprid	6.69	223.0	126.1	Positive	18	223.0	90.1	Positive	35
Avermectin B1a	17.70	890.5	307.2	Positive	12	890.5	305.1	Positive	8
						890.5	145.0	Positive	24
Chlorantraniliprole	10.03	483.9	452.9	Positive	16	483.9	285.9	Positive	8
Clothianidin	6.66	250.0	169.0	Positive	8	250.0	131.9	Positive	8
Dimethoate	6.48	230.0	125.0	Positive	24	230.0	47.0	Positive	56
						230.0	199.0	Positive	4
Etofenprox	18.66	394.2	177.2	Positive	13	394.2	359.2	Positive	12
						394.2	107.1	Positive	45
Fenpyroximate	16.43	422.2	366.2	Positive	12	422.2	135.0	Positive	36
						422.2	107.0	Positive	64
Flonicamid	5.84	230.0	174.0	Positive	15	230.0	148.1	Positive	24
						230.0	98.1	Positive	48
Hexythiazox	16.45	353.1	168.1	Positive	24	353.1	227.9	Positive	8
						353.1	116.1	Positive	56
Imidacloprid	6.57	256.0	175.1	Positive	22	256.0	209.1	Positive	12
						256.0	84.0	Positive	20
Indoxacarb	14.30	528.0	203.0	Positive	40	528.0	293.0	Positive	9
						528.0	149.9	Positive	25
Methiocarb	11.03	226.1	169.1	Positive	4	226.1	121.1	Positive	15
Pirimicarb	8.20	239.1	72.1	Positive	20	239.1	182.2	Positive	13
						239.1	109.1	Positive	32
Pymetrozine	5.66	218.0	105.0	Positive	20	218.0	79.0	Positive	49
						218.0	78.1	Positive	45
Spinosad A	13.64	732.5	142.1	Positive	30	732.5	98.0	Positive	45
Spinosad D	14.58	746.5	142.1	Positive	15	746.5	98.0	Positive	55
Tebufenozide	12.94	353.2	133.0	Positive	16	353.2	297.1	Positive	0
						353.2	105.1	Positive	44
Thiacloprid	6.86	253.0	126.0	Positive	20	253.0	99.1	Positive	44
						253.0	90.1	Positive	44
Thiamethoxam	5.98	292.0	211.1	Positive	10	292.0	181.1	Positive	28
						292.0	131.9	Positive	40

Table A9/3. (cont.). MRM transitions of analytes in positive and negative ion mode method.

Substance	Quantifier					Qualifiers			
	**Retention time (minutes)**	**Precursor ion**	**Product ion**	**Ion polarity**	**Collision energy (eV)**	**Precursor ion**	**Product ion**	**Ion polarity**	**Collision energy (eV)**
Biocides									
Fipronil desulfinyl	11.70	386.8	350.8	Negative	20	386.8	350.8	Negative	20
Fipronil	12.77	435.0	333.0	Negative	12	435.0	250.0	Negative	28
Fipronil sulfone	13.27	450.8	218.8	Negative	40	450.8	243.8	Negative	70

Figures:

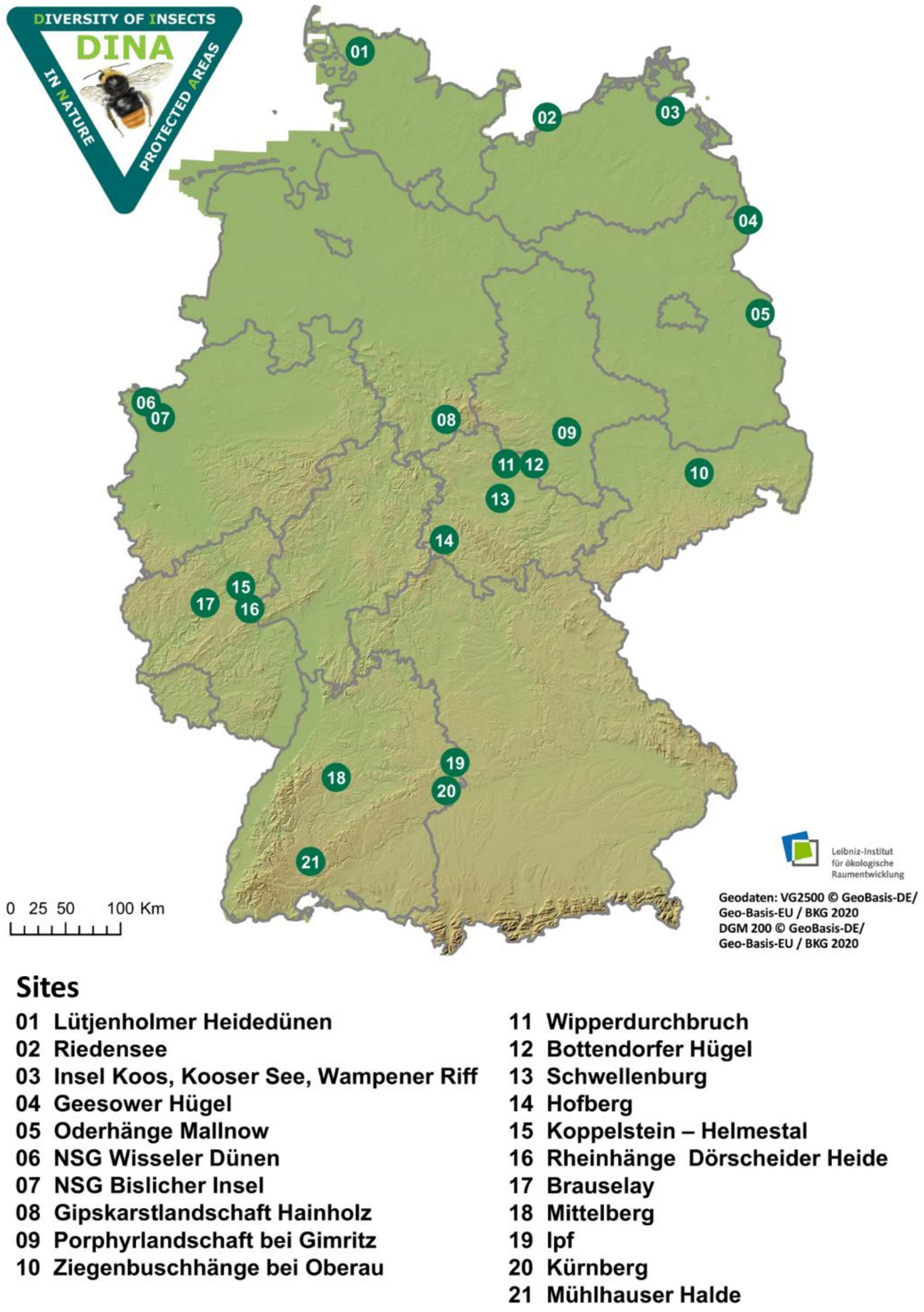

Figure A1 Map with location of study sites in Germany (Created by Lisa Eichler/Hanna Poglitsch using Esri ArcMap 10.8.1 (https://support.esri.com/de/products/desktop/arcgis-desktop/arcmap/10-8).

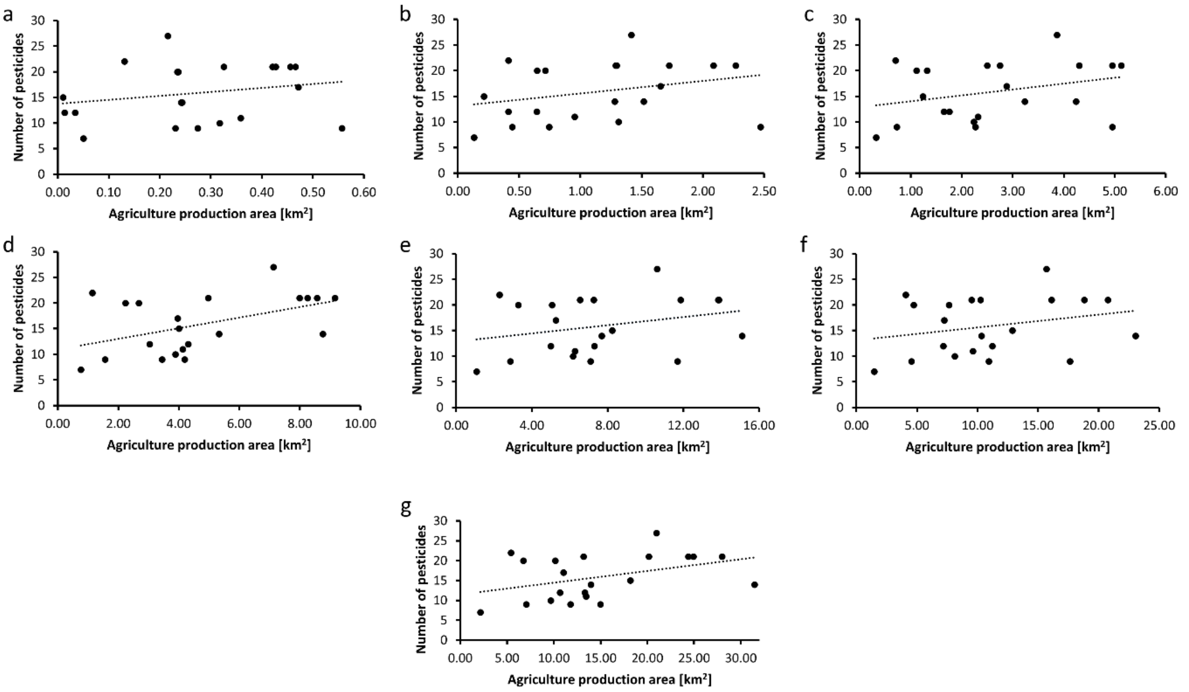

Figure A2. Correlation between the number of pesticide residues and the amount of arable land in the respective radii from 500-3500 m (a=500 m, b=1000m, c=1500 m, d=2000 m, e=2500 m, f=3000 m, and g=3500 m, for values see also Table A6). All 21 sites included.

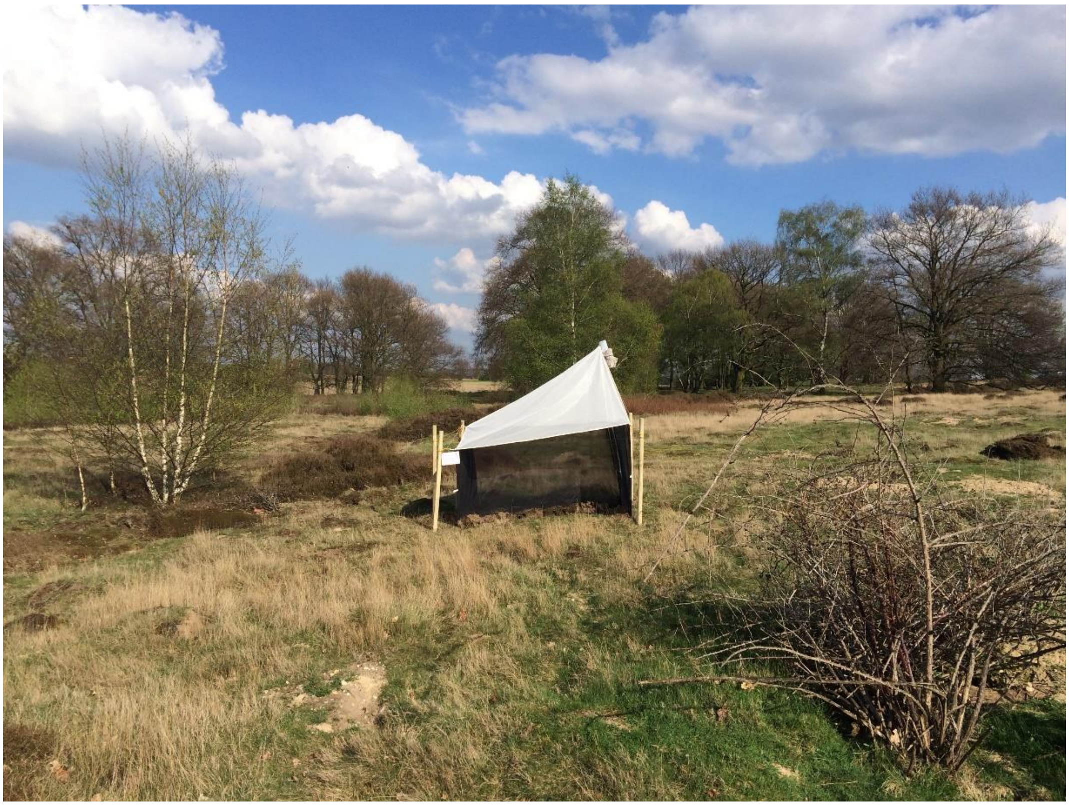

A

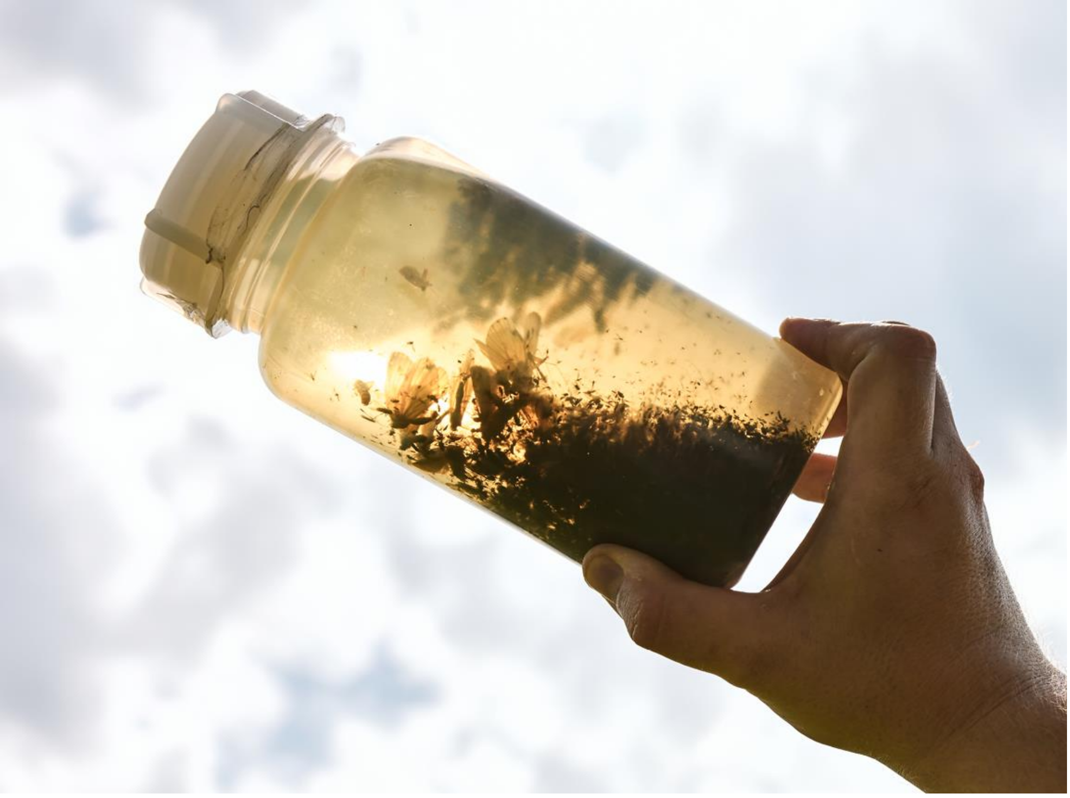

B

Figure A3 ***A:*** *Malaise trap for collecting flying insects. Collecting bottle can be seen in the top right corner (Martin Sorg).* ***B:*** *Collecting bottle with ethanol and insect sample after running for 14 days (Thomas Hörren) (© EVK, Creative Commons 4.0).*

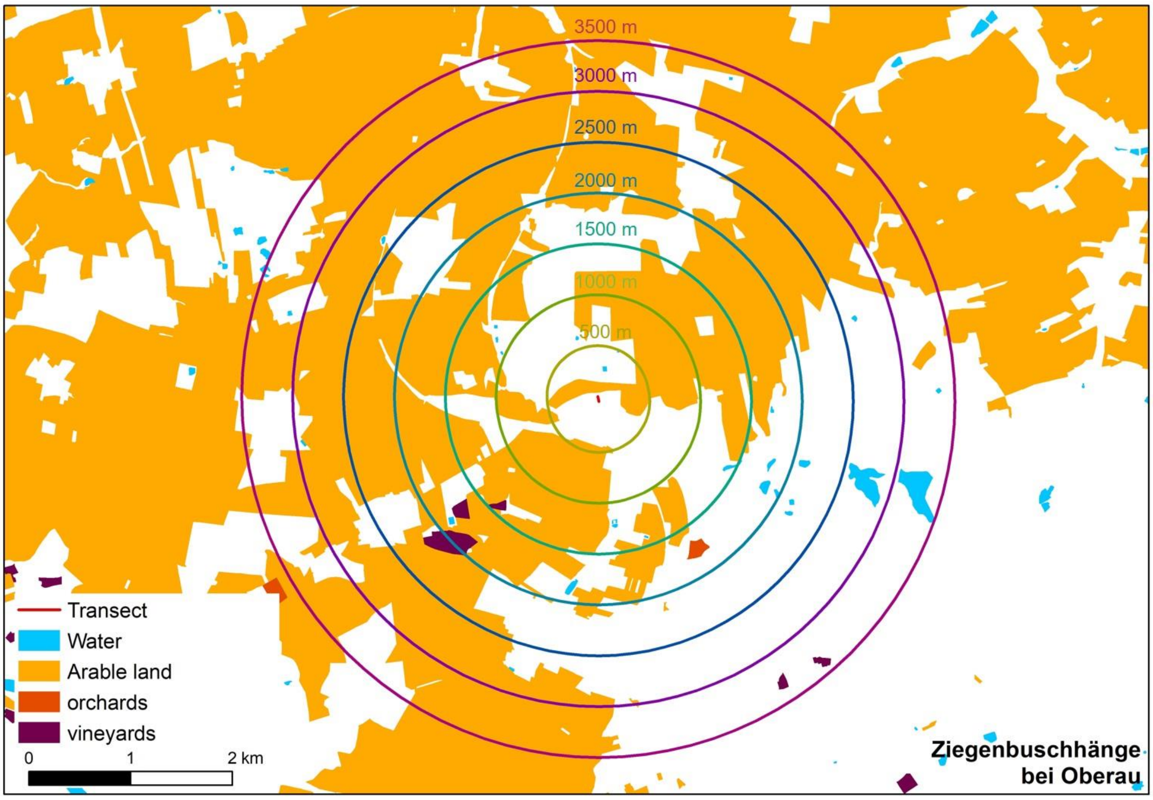

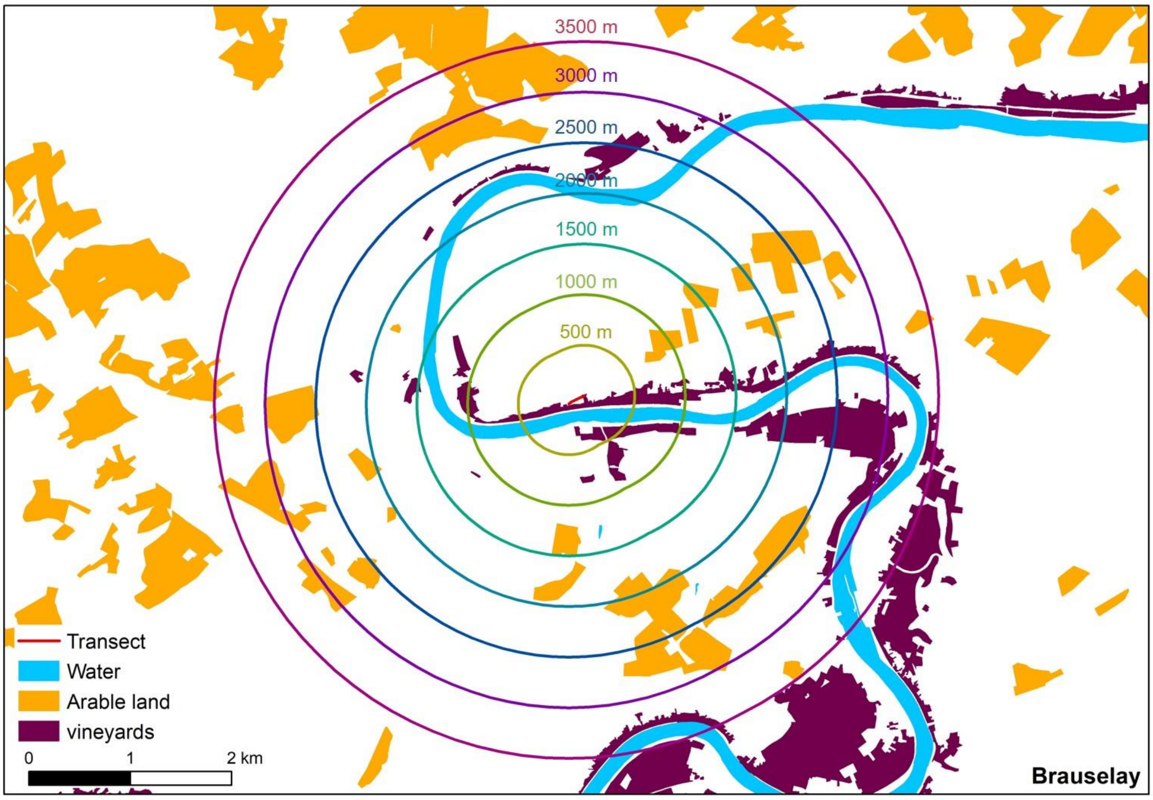

Figure A4 Calculation of buffer radii at sites. ***A:*** *site 10 Ziegenbuschhänge bei Oberau* ***B:*** *site 17 Brauselay, situated in a wine growing area. (Created by Lisa Eichler using Esri ArcMap 10.8.1 (https://support.esri.com/de/products/desktop/arcgis-desktop/arcmap/10-8).*
